# Clinical significance of HER2 in urothelial carcinoma and analysis of its correlation with glycolytic metabolic characteristics

**DOI:** 10.3389/fmolb.2024.1521889

**Published:** 2024-12-09

**Authors:** Andong Guo, Chenrui Wu, Jishuang Cao, Kejia Zhu, Sentai Ding

**Affiliations:** ^1^ Department of Urology, Shandong Provincial Hospital Affiliated to Shandong First Medical University, Jinan, Shandong, China; ^2^ Department of Urology, Shandong Provincial Hospital, Cheeloo College of Medicine, Shandong University, Jinan, Shandong, China

**Keywords:** urothelial carcinoma, HER2, glycolytic metabolism, lactate dehydrogenase, therapeutic targets

## Abstract

**Objective:**

This study aimed to explore the clinical relevance of Human Epidermal Growth Factor Receptor 2 (HER2) in urothelial carcinoma (UC) and its association with glycolytic metabolic markers, insulin resistance, and beta-cell function, shedding light on potential therapies targeting both HER2 pathways and cancer metabolism.

**Methods:**

In this retrospective analysis, 237 UC patients from the Departments of Urology and Pathology at Shandong Provincial Hospital were examined. From 1 January 2023, to 1 October 2024, patients underwent HER2 testing using immunohistochemistry (IHC). We investigated the relationships between HER2 expression and metabolic indicators such as the Homeostatic Model Assessment for insulin resistance (HOMA-IR), beta-cell function (HOMA-β), the triglyceride-glucose (TyG) index, and lactate dehydrogenase (LDH) levels. HER2 status was determined using a standardized scoring system from the 2021 Clinical Pathological Expert Consensus on HER2 Testing in UC, China. Statistical analysis followed CDC guidelines, using multivariate logistic regression to assess the independent impacts of HER2 on metabolic traits.

**Results:**

Of the 237 evaluated UC samples, 87.76% exhibited positive HER2 expression. A significant correlation was found between positive HER2 status, advanced tumor stages, and increased LDH levels, suggesting a link between HER2 expression and heightened glycolytic activity. No significant relationships were observed between HER2 status and TyG levels, HOMA-IR, or HOMA-B. Subgroup analyses confirmed the consistency of the relationship between HER2 expression and LDH levels across different demographics and lifestyle factors.

**Conclusion:**

Our findings confirm the significant role of HER2 as a prognostic marker and therapeutic target in UC. The association of HER2 positivity with advanced tumor stages and high LDH levels underscores its complex involvement in disease progression. This study highlights the need to explore HER2’s biological mechanisms further and pursue combined therapeutic strategies.

## Introduction

Urothelial carcinoma (UC) is a significant global health concern, primarily affecting the urinary bladder but also found in the upper urinary tract and urethra (Author Anonymous, 2021). It is known for its aggressive behavior, frequent recurrence, and potential for metastasis, imposing a substantial burden on patients and healthcare systems. UC incidence varies geographically, with higher rates observed in industrialized nations, likely due to increased exposure to environmental and occupational carcinogens, including tobacco smoke, aromatic amines, and polycyclic aromatic hydrocarbons (Bray et al., 2024). Despite progress in diagnostic and therapeutic methods, the prognosis for metastatic UC remains poor, with a 5-year survival rate below 5%. This highlights the critical need for improved understanding, early detection, and innovative treatment strategies (Cathomas et al., 2022). The treatment landscape for UC has significantly shifted over the last decade from conventional chemotherapy to more personalized strategies. A key advancement is the identification of HER2 as a molecular target, which has enhanced our understanding of UC and spurred the development of targeted therapies. Recent studies highlight the effectiveness of HER2-targeted therapies like disitamab vedotin (RC48-ADC) (Sheng et al., 2021). This antibody-drug conjugate shows potential in treating HER2-positive metastatic UC patients resistant to standard therapies, illustrating a broader trend towards precision oncology where treatments are tailored based on molecular profiling.

An area of growing interest is the role of Human Epidermal Growth Factor Receptor 2 (HER2) in UC. HER2 is a transmembrane glycoprotein receptor with intrinsic tyrosine kinase activity, known for regulating cellular proliferation, differentiation, and survival (Schechter et al., 1985; van der Geer et al., 1994). Aberrant HER2 expression, primarily due to gene amplification or mutation, has been implicated in developing various epithelial cancers, including breast and gastric cancers (Moasser, 2007). In UC, HER2 overexpression has been reported at varying frequencies, ranging from 9% to 61% (Chen et al., 2021). Some studies have suggested a link between HER2 overexpression and poor prognosis in UC, while others have found no such correlation (Jimenez et al., 2001; Moktefi et al., 2018). These discrepancies may stem from differences in HER2 assessment methods, patient populations, and treatment regimens (Bellmunt et al., 2015). The role of HER2 in cancer biology extends beyond its classical function as a growth factor receptor. Recent research has revealed its complex interactions with tumor microenvironment, immune response, and metabolic reprogramming (Shi et al., 2021). Studies have shown that HER2 signaling influences tumor immune evasion mechanisms and metabolic adaptation, particularly through the PI3K/AKT/mTOR pathway (Kavarthapu et al., 2021). Understanding these broader biological implications is crucial for developing more effective therapeutic strategies.

Recent studies have indicated a strong link between HER2 overexpression and metabolic reprogramming in various cancers (Premaratne et al., 2024), particularly with altered glucose metabolism. One notable adaptation is the Warburg effect, where cancer cells preferentially use glycolysis, even in the presence of oxygen, to support their rapid growth and proliferation (Zhang et al., 2012). Beyond enhanced glycolytic activity, insulin resistance and changes in pancreatic beta-cell function have also been associated with tumor metabolic reprogramming (Chiefari et al., 2021; Javeed et al., 2015). These metabolic alterations are critical for meeting the elevated energy needs of proliferating cancer cells and providing the anabolic intermediates necessary for biosynthesis.

Emerging evidence suggests that HER2 influences the metabolic phenotype of cancer cells by regulating vital glycolytic enzymes, insulin signaling pathways, and beta-cell function, thereby enhancing glucose uptake and metabolism to support cancer cell survival and growth, even under challenging conditions like hypoxia (Wolpin et al., 2013; Yuan et al., 2023; Bollig-Fischer et al., 2011). Understanding the link between HER2 overexpression and these broader glycolytic metabolic adaptations in UC is essential for identifying new therapeutic targets to disrupt these oncogenic and metabolic pathways.

This study aims to investigate the clinical significance of HER2 expression in UC, focusing on its impact on glycolytic metabolic reprogramming, insulin resistance, and beta-cell activity. By analyzing the correlation between HER2 expression and metabolic markers of glycolytic reprogramming, insulin resistance, and beta-cell function, we seek to elucidate the role of HER2 in metabolic adaptation in UC. This could provide insight into potential therapeutic approaches targeting both HER2 signaling and cancer metabolism.

## Materials and methods

### The study population

This retrospective study, approved by the Research Ethics Committee of Shandong Provincial Hospital affiliated with Shandong First Medical University (Jinan, China; No. 2024-046), was exempt from informed consent requirements due to the use of anonymized and confidential patient data. It included specimens from UC collected through various surgical interventions—transurethral resection of bladder tumors, partial cystectomy, radical cystectomy, segmental ureterectomy, and total ureteropelvic resection—conducted between January 2023 and October 2024. Patients with significant cardiovascular, hepatic, or renal dysfunctions, which could compromise survival or data interpretation, were excluded. Clinicopathological data retrieved from the hospital’s electronic medical database included age, gender, year of surgery, primary tumor site, stage, grade, growth pattern, focality, presence of metastasis, history of diabetes, smoking and drinking habits, and hematological and glucose metabolism indicators. Tumor staging and grading were carried out according to the 2017 TNM system and the 2016 WHO criteria, respectively.

### Definition

Homeostatic model assessments for insulin resistance (HOMA-IR) and β-cell function (HOMA-B) were conducted using the following formulas: HOMA-IR was calculated as [fasting glucose (FBG, nmol/L) × fasting insulin (mU/mL)]/22.5, whereas HOMA-B was derived from [20 × fasting insulin (μU/mL)]/[FBG (mmol/L) − 3.5] (Mazidi et al., 2018). The triglyceride-glucose (TyG) index was determined using the equation ln [(fasting triglycerides (TG, mg/dL) × FBG (mg/dL))/2] (Mazidi et al., 2018). Lactate dehydrogenase (LDH), an essential enzyme reflecting glycolytic activity, was measured through the enzyme rate method to evaluate serum LDH levels in all adult participants at baseline.

### HER2 immunohistochemistry

HER2 Expression Assessment: UC specimens were fixed within 1 hour of collection using 10% neutral formalin, embedded in paraffin, and sectioned at a thickness of 4 μm. Sections were incubated overnight with a primary antibody at 4°C, washed three times with phosphate-buffered saline, and then treated with a secondary antibody at 37°C for 30 min. Streptavidin-biotin complex and diaminobenzidine solutions were applied sequentially, followed by hematoxylin counterstaining (Hicks and Kulkarni, 2008).

Following preparation, HER2 immunohistochemistry (IHC) analysis was performed by the 2021 Clinical Pathological Expert Consensus on HER2 Testing in UC, China (Tumor Pathology Committee of Chinese Anti-Cancer AssociationExpert Committee on Urothelial Carcinoma of Chinese Society of Clinical Oncology, 2021). The scoring criteria were as follows: score 0 (no staining or <10% of cells with weak incomplete membrane staining), score 1 + (≥10% of cells with weak incomplete membrane staining), score 2 + (≥10% of cells with weak-to-moderate complete membrane staining or <10% of cells with complete solid membrane staining), and score 3 + (≥10% of cells with intense complete membrane staining). HER2 overexpression was defined by IHC scores of 2+ and 3+. Each slide was independently assessed by two senior pathologists, with any discrepancies resolved by a third pathologist.

### Statistical analysis

Statistical analyses were conducted following CDC guidelines. For variables conforming to a normal distribution, means and standard deviations were calculated, and group differences were assessed using the Student's t-test; for variables that did not adhere to a normal distribution or exhibited significant variance discrepancies, medians, and interquartile ranges were reported, with differences evaluated using the Mann-Whitney U test. Categorical variables were expressed as percentages and intergroup differences were analyzed using the chi-square test.

Separate multivariate logistic regression models were employed to explore the independent effects of HER2 expression on glycolytic metabolic characteristics. Model 1 provided a baseline analysis without adjustments, offering an initial overview of the data. Model 2 included adjustments for age and diabetes status, addressing essential demographic factors that could influence the outcomes. Model 3, the most comprehensive model, further adjusted for additional variables, including alcohol consumption, smoking status, and medication use. For variables demonstrating significant differences, subgroup analyses were conducted to examine further the association between HER2 expression and glycolytic metabolic characteristics, stratified by age, diabetes status, and history of smoking and alcohol consumption. Statistical significance was defined as *p* < 0.05.

All analyses were performed using R version 4.3.0 and Empower software (X&Y Solutions, Inc., Boston, MA, United States).

## Results

### Baseline of clinical patients

In this study, we examined the clinical characteristics of patients diagnosed with tumors in the bladder, ureter, and renal pelvis. We included 174 patients with bladder tumors, 39 with ureteral tumors, and 24 with renal pelvis tumors (Table 1). The average ages were 66.40 ± 10.61 years for bladder tumor patients, 68.59 ± 8.65 years for ureteral, and 69.50 ± 9.29 years for renal pelvis tumors. No significant age differences were observed across these groups (*p* = 0.351). There were no significant differences in LDH levels, HOMA-IR, HOMA-β, or TYG among the three groups (*p* = 0.105, *p* = 0.388, *p* = 0.073, and *p* = 0.294, respectively).

**TABLE 1:** Baseline clinicopathological features of the analyzed cohort.

Characteristic	Bladder	Ureteral	Renal pelvis	*p*-value
N	174	39	24	
Age/years	66.40 ± 10.61	68.59 ± 8.65	69.50 ± 9.29	0.351
Tumor size/cm	2.73 ± 1.57	3.08 ± 1.86	3.60 ± 1.89	0.039
LDH	171.63 ± 16.35	173.88 ± 14.75	176.92 ± 17.83	0.105
HOMA-IR	3.84 ± 3.01	4.40 ± 3.93	4.05 ± 4.60	0.388
HOMA-β	120.46 ± 84.13	100.76 ± 69.95	82.18 ± 50.34	0.073
TYG	8.95 ± 0.69	8.80 ± 0.67	8.78 ± 0.65	0.294
Gender				<0.001
Male	27 (15.52%)	17 (43.59%)	17 (70.83%)	
Female	147 (84.48%)	22 (56.41%)	7 (29.17%)	
Muscle Invasion				0.002
MIUC	48 (27.59%)	2 (5.13%)	2 (8.33%)	
NMIUC	126 (72.41%)	37 (94.87%)	22 (91.67%)	
HER2 (+/−)				0.061
-	51 (29.31%)	19 (48.72%)	9 (37.50%)	
+	123 (70.69%)	20 (51.28%)	15 (62.50%)	
pN				0.011
N1	166 (95.40%)	35 (89.74%)	19 (79.17%)	
N0	8 (4.60%)	4 (10.26%)	5 (20.83%)	
Metastasis				0.362
No	169 (97.13%)	36 (92.31%)	23 (95.83%)	
Yes	5 (2.87%)	3 (7.69%)	1 (4.17%)	
Grade				0.014
Low-grade	50 (28.74%)	3 (7.69%)	4 (16.67%)	
High-grade	124 (71.26%)	36 (92.31%)	20 (83.33%)	
Stage				<0.001
Ⅰ	108 (62.07%)	16 (41.03%)	6 (25.00%)	
Ⅱ	27 (15.52%)	11 (28.21%)	3 (12.50%)	
Ⅲ	27 (15.52%)	6 (15.38%)	8 (33.33%)	
IV	12 (6.90%)	6 (15.38%)	7 (29.17%)	

Abbreviations: N, number; pN, primary lymph node stage; MIBC, muscle-invasive urothelial cancer; NMIBC, non-muscle invasive urothelial cancer. Statistical significance was defined as *p*< 0.05.

There was a significant difference in gender distribution among the tumor sites (*p* < 0.001), with a more significant percentage of males in the ureteral (43.59%) and renal pelvis (70.83%) groups compared to the bladder group (15.52%), which was predominantly female (84.48%). Muscle invasion varied significantly by tumor site (*p* = 0.002), being less common in ureteral (5.13%) and renal pelvis tumors (8.33%) than in bladder tumors (27.59%).

The HER2 status approached statistical significance (*p* = 0.061), with higher percentages of HER2-negative cases in the ureteral (48.72%) and renal pelvis (37.50%) groups compared to the bladder group (29.31%). Lymph node involvement, as indicated by pN status, differed significantly across the groups (*p* = 0.011), with fewer N0 cases in the renal pelvis (20.83%) relative to the bladder (4.60%) and ureteral (10.26%) groups. The incidence of metastasis was low and did not differ significantly among the groups (*p* = 0.362). Grade and stage distributions were also significantly different, with a higher proportion of high-grade and advanced-stage tumors in the ureteral and renal pelvis cancer groups compared to bladder cancer (*p* = 0.014 and *p* < 0.001, respectively). These findings underscore that tumor characteristics such as size, muscle invasion, HER2 status, lymph node involvement, metastasis, and grade vary notably by tumor site, influencing potential treatment approaches and patient prognosis.

### HER2 expression in UC and its correlation with clinicopathological characteristics

**FIGURE 1:**
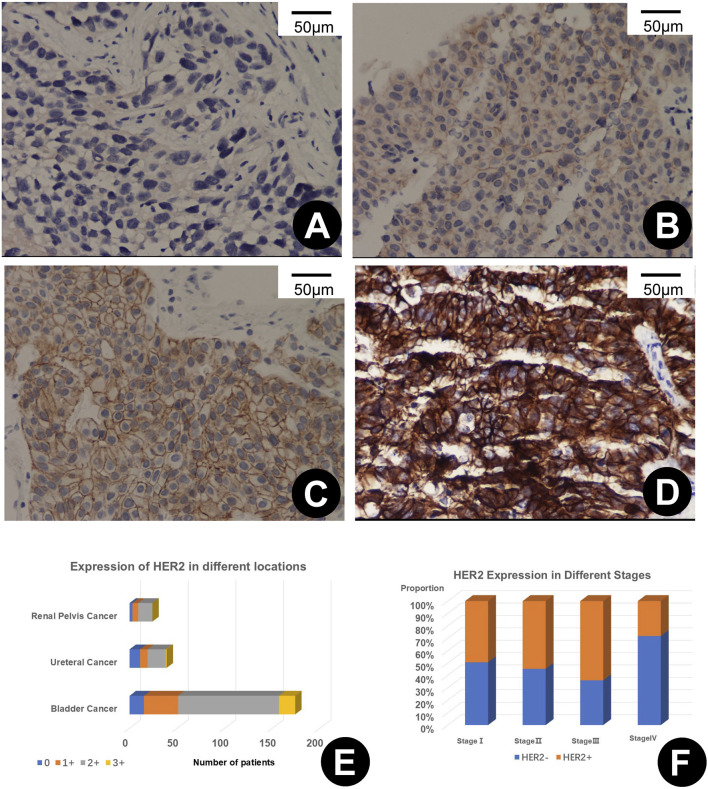
**(A–D)** depict varying levels of HER2 immunohistochemical (IHC) expression in urothelial carcinoma, scored as 0+ **(A)**, 1+ **(B)**, 2+ **(C)**, and 3+ **(D)**, each shown at 200x magnification. **(E)** illustrates the variability of HER2 expression across different primary sites of urothelial carcinoma. **(F)** shows the rates of HER2 positivity at different stages of the disease.

In the comprehensive evaluation of HER2 protein expression in 237 UC tissues, positive expression was observed in 208 samples (87.76%). Expression was stratified as 1+ in 50 samples (21.1%), 2+ in 140 samples (59.07%), and 3+ in 18 samples (7.59%). Analysis of 174 bladder tumors revealed HER2 expression in 159 specimens (91.38%), distributed as 36 with a 1+ score (20.69%), 106 with a 2+ score (60.92%), and 17 with a 3+ score (9.77%). Among 28 ureteral tumors, 39 exhibited HER2 expression (71.79%), with eight at 1+ (20.51%), 19 at 2+ (48.72%), and a single case at 3+ (2.56%). Of the 24 renal pelvis tumors studied, 21 showed HER2 positivity (87.5%), with scoring of 1+ in 6 cases (25.00%) and 2+ in 15 cases (62.50%), as depicted in Figures 1E, F.

In our comparative analysis of patients with HER2-negative (HER2-) and HER2-positive (HER2+) tumors, we observed no significant differences in the age distribution and tumor size between the two groups (Table 2). The mean age was 65.72 ± 10.08 years for HER2-patients and 67.75 ± 10.25 years for HER2+ patients (*p* = 0.151), while the mean tumor size was 3.12 ± 1.92 cm for HER2-patients and 2.75 ± 1.53 cm for HER2+ patients (*p* = 0.103). Gender distribution was also similar, with 29.11% of HER2-patients and 24.05% of HER2+ patients being male, 70.89% of HER2-patients, and 75.95% of HER2+ patients being female (*p* = 0.401).

**TABLE 2:** Relationship between HER2+ and some clinicopathological features of UC.

Characteristic	HER2-	HER2+	*p*-value
N	79	158	
Age/years	65.72 ± 10.08	67.75 ± 10.25	0.151
Tumor size/cm	3.12 ± 1.92	2.75 ± 1.53	0.103
Gender			0.401
Male	23 (29.11%)	38 (24.05%)	
Female	56 (70.89%)	120 (75.95%)	
Muscle Invasion			0.824
MIUC	18 (22.78%)	34 (21.52%)	
NMIUC	61 (77.22%)	124 (78.48%)	
Primary site			0.061
Bladder	51 (64.56%)	123 (77.85%)	
Ureter	19 (24.05%)	20 (12.66%)	
Renal pelvis	9 (11.39%)	15 (9.49%)	
pN			0.213
N1	71 (89.87%)	149 (94.30%)	
N0	8 (10.13%)	9 (5.70%)	
Metastasis			1.000
No	76 (96.20%)	152 (96.20%)	
Yes	3 (3.80%)	6 (3.80%)	
Grade			0.368
Low-grade	24 (30.38%)	33 (20.89%)	
High-grade	55 (69.62%)	125 (79.11%)	
Stage			0.037
Ⅰ	44 (55.70%)	86 (54.43%)	
Ⅱ	12 (15.19%)	29 (18.35%)	
Ⅲ	9 (11.39%)	32 (20.25%)	
IV	14 (17.72%)	11 (6.96%)	

Abbreviations: N, number; pN, primary lymph node stage; MIBC, muscle-invasive urothelial cancer; NMIBC, non-muscle invasive urothelial cancer; HER2, human epidermal growth factor receptor 2. Statistical significance was defined as *p* < 0.05.

Muscle invasion, as measured by the presence of muscle-invasive UC (MIUC) and non-muscle-invasive UC (NMIUC), did not differ significantly between HER2-and HER2+ patients (*p* = 0.824), with 22.78% of HER2-patients and 21.52% of HER2 + patients exhibiting MIUC. The primary tumor site distribution was nearly significant, with a higher proportion of HER2-patients having bladder tumors (64.56%) compared to HER2+ patients (77.85%) and a lower proportion of HER2-patients having ureteral (24.05%) and renal pelvis (11.39%) tumors compared to HER2+ patients (12.66% and 9.49%, respectively) (*p* = 0.061).

Lymph node involvement, indicated by pN status, was not significantly different between HER2-and HER2+ patients (*p* = 0.213), with 89.87% of HER2-patients and 94.30% of HER2+ patients being N1. Metastasis was equally distributed between the two groups, with 3.80% of patients in each group having metastatic disease (*p* = 1.000).

The grade distribution neared significance, with a higher proportion of high-grade tumors observed in the HER2-positive group (79.11%) compared to the HER2-negative group (69.62%), although not statistically significant (*p* = 0.368). In contrast, stage distribution differed significantly between the groups (*p* = 0.037), with HER2-positive patients showing a greater prevalence of advanced-stage tumors (stages III and IV) and a reduced proportion of stage I tumors relative to HER2-negative patients. These results indicate that HER2 status may be linked to tumor aggressiveness and stage, potentially affecting treatment decisions and prognosis in UC.

### Association between HER2 expression and glycometabolic characteristics

In analyzing HER2 expression’s independent effects on glycolytic metabolic characteristics, multivariate logistic regression models were employed to evaluate the associations (Table 3). Initially, HER2 expression was significantly correlated with LDH levels, exhibiting a positive beta coefficient of 0.40 (95% CI: 0.07, 0.73) and a *p*-value of 0.0186. This finding indicates a potential link between HER2 expression and increased LDH levels in the unadjusted model. However, no significant association was found with TYG levels, as indicated by a non-significant *p*-value of 0.5004 and a beta coefficient of 2.84 (95% CI: −5.40, 11.07). Similarly, insulin resistance indices, such as HOMA-IR and HOMA-β, did not show statistical significance in Model 1, with *p*-values of 0.0685 and 0.1790, respectively.

**TABLE 3:** Association between HER2 expression and glycometabolic characteristics.

Exposure	Outcome	Model 1: β (95% CI) *p*	Model 2: β (95% CI) *p*	Model 3: β (95% CI) *p*
HER2	LDH	0.40 (0.07,0.73) 0.0186	0.35 (0.02,0.69) 0.0402	0.36 (0.04,0.68) 0.0272
HER2	TYG	2.84 (−5.40, 11.07) 0.5004	1.64 (−6.70, 9.98) 0.7000	1.13 (−7.20, 9.46) 0.7901
HER2	HOMA-IR	−1.81 (−3.76, 0.13) 0.0685	−1.75 (−3.72, 0.22) 0.0826	−1.83 (−3.76, 0.11) 0.0662
HER2	HOMA-β	−24.10 (−59.10, 10.91) 0.1790	−13.84 (−49.00, 21.33) 0.4416	−13.31 (−48.47, 21.85) 0.4590

Abbreviations: HER2, human epidermal growth factor receptor 2; HOMA-IR, homeostatic model assessments for insulin resistance; HOMA-β, Homeostatic model assessments for β-cell function; TyG, triglyceride-glucose index; LDH, Lactate dehydrogenase; OR, odds ratio; CI, confidence interval; Statistical significance was defined as *p* < 0.05.

Model 2 incorporated adjustments for age and diabetes status. Within this model, the significant relationship between HER2 expression and LDH levels persisted, evidenced by a beta coefficient of 0.35 (95% CI: 0.02, 0.69) and a *p*-value of 0.0402. This suggests that the association withstands adjustments for essential demographic factors. The non-significant results for TYG, HOMA-IR, and HOMA-β observed in Model 1 continued in Model 2, with *p*-values of 0.7000, 0.0826, and 0.4416, respectively.

The most extensive Model 3 further adjusted for alcohol consumption, smoking status, and medication use. Despite these comprehensive adjustments, the significant association of HER2 expression with LDH levels was sustained, with a beta coefficient of 0.36 (95% CI: 0.04, 0.68) and a *p*-value of 0.0272. The relationships with TYG, HOMA-IR, and HOMA-β remained non-significant in Model 3, with *p*-values of 0.7901, 0.0662, and 0.4590, respectively. These outcomes reinforce that HER2 expression is independently associated with LDH levels, irrespective of potential confounders. No significant associations were observed with TYG, HOMA-IR, and HOMA-β across all models.

### Subgroup analysis

**FIGURE 2:**
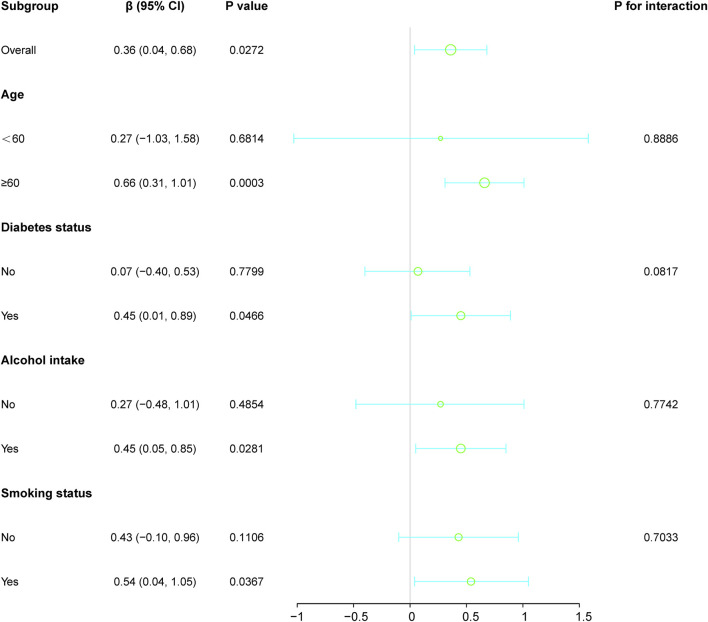
Subgroup analyses investigated the relationship between HER2 expression and LDH levels, considering factors such as age, diabetes status, alcohol intake, and smoking habits. Except for the stratification component, each factor was adjusted for all other variables (age, diabetes status, alcohol intake, and smoking habits).

Subgroup analyses investigated the relationship between HER2 expression and LDH levels, considering age, diabetes status, alcohol intake, and smoking habits (Figure 2). This revealed notable variations across different demographics. Overall, a significant positive association was evident in the general population, indicated by a beta coefficient of 0.36 (95% CI: 0.04, 0.68; *p*-value: 0.0272).

Stratification by age showed stronger associations in individuals aged 60 and above, with a beta coefficient of 0.66 (*p*-value: 0.0003), in contrast to those under 60, where the association was insignificant (beta coefficient: 0.27; *p*-value: 0.6814). Similarly, diabetic individuals demonstrated a significant positive relationship (beta coefficient: 0.45; p-value: 0.0466), which was not observed in non-diabetic patients.

Among lifestyle factors, drinkers and smokers also showed significant correlations (beta coefficients: 0.45 and 0.54, respectively), whereas no significant associations were found between non-drinkers and non-smokers. The interaction terms across all subgroup factors indicated no significant modifications (*p* > 0.05), confirming a consistent association between HER2 expression and LDH levels across the examined demographic and lifestyle variables.

## Discussions

In this study, we observed a HER2 overexpression rate of 66.66% in UC, with significant associations between HER2 positivity and specific clinicopathological characteristics. Notably, HER2 expression was considerably higher in bladder tumors than in ureteral and renal pelvis tumors, suggesting a more critical role of HER2 overexpression in bladder cancer biology. Additionally, our analysis identified a significant correlation between HER2 expression and advanced tumor stage, pointing to a potential relationship between HER2 positivity and increased tumor aggressiveness. Importantly, HER2 expression was independently linked to elevated levels of LDH, a key marker of glycolytic metabolism, even after adjusting for demographic and lifestyle factors.

Recent research increasingly highlights the critical role of genetic abnormalities in the etiology and pathogenesis of UC, with a notable focus on the HER2 protein (Moasser, 2007). The involvement of HER2 in tumorigenesis is multifaceted and may engage various signaling pathways, such as PI3K/AKT/mTOR, Ras/Raf/MAPK, and STAT (Shi et al., 2021; Kavarthapu et al., 2021; Zhang et al., 2023). These pathways are activated by heterodimerization of HER family receptors and autophosphorylation of their intracellular tyrosine kinase domains (Galogre et al., 2023). Research by Tan et al. indicates that HER2 overexpression may undermine BCG therapy efficacy by activating the PI3K-Akt pathway and upregulating antiapoptotic proteins (Tan et al., 2024). In non-muscle invasive bladder cancer patients, HER2 overexpression (IHC score 2/3+) is associated with higher rates of recurrence and progression, with 5-year recurrence-free survival and progression-free survival rates of just 19.0% and 58.2%, respectively. These results suggest that HER2 expression could be a vital biomarker for assessing tumor development and predicting BCG treatment responses.

The high rate of HER2 expression we observed, at 66.66%, underscores its potential significance in UC biology. This finding aligns with Zhou et al., who reported a 44% positivity rate (Zhou et al., 2023). However, our results encompass a broader range than the 9%–61% reported by Scherrer et al., highlighting the heterogeneity in HER2 positivity rates and underscoring the need for standardized testing protocols (Scherrer et al., 2022).

HER2 overexpression is particularly pronounced in bladder tumors as opposed to those in the ureter and renal pelvis, suggesting varying dependencies on HER2 signaling across UC subtypes. Our findings align with Bellmunt et al., who noted similar variations in HER2 positivity between Spanish and Greek cohorts, indicating a stable prevalence of HER2 overexpression across different ethnicities (Bellmunt et al., 2015). Moreover, our results support the molecular subtype analysis by Tan et al., which identified distinct genomic landscapes between the bladder and upper tract UC (Tan et al., 2019).

Our study further establishes a link between HER2 positivity and advanced tumor stages, indicative of the aggressive nature of HER2-positive tumors, as also demonstrated by Soria et al. (2017). This association suggests that HER2 expression could serve as a marker of tumor progression and aggressiveness in UC, having significant implications for patient stratification and treatment allocation. Additionally, the correlation aligns with findings from Zhou et al., linking HER2 expression to aggressive disease (Zhou et al., 2023), and is supported by Fleischmann et al., who reported a higher frequency of HER2 amplification in lymph node metastases compared to primary tumors (Fleischmann et al., 2011).

We also observed an independent association between HER2 expression and elevated levels of LDH, which is markedly upregulated in various cancers, including lymphoma, UC, renal cell carcinoma, and melanoma (Claps et al., 2022; Miholjcic et al., 2023). LDH facilitates a shift toward aerobic glycolysis, creating hypoxic and acidic conditions within the tumor microenvironment that support tumor growth, survival, invasion, migration, metastasis, and angiogenesis, ultimately contributing to the tumor’s immune evasion (Kocianova et al., 2022; Sharma et al., 2022; Nimmakayala et al., 2021).

We found no significant correlations between HER2 and other metabolic markers such as TYG, HOMA-IR, and HOMA-β. Recent studies have presented mixed findings on the relationship between HER2 and metabolic parameters in cancer. For instance, Zhang et al. found that higher TYG index levels were linked to poorer outcomes in gastric cancer. The absence of a similar correlation in our study may suggest that HER2-mediated metabolic reprogramming in urothelial carcinoma operates through mechanisms distinct from those involved in triglyceride-glucose metabolism. Furthermore, Zhu et al. observed that oncogene activation, including HER2, could lead to a metabolic shift resulting in insulin independence in breast cancer cells. Our results, which show no significant associations between HER2 and insulin-related markers, might reflect tissue-specific variations in how HER2 affects insulin sensitivity and β-cell functionality across different types of cancer.

HER2-targeted therapies offer considerable potential in UC, particularly with antibody-drug conjugates (ADCs) such as RC48-ADC (Hong et al., 2023). This treatment is a viable and safe option for patients with HER2-positive mUC after first-line chemotherapy. RC48-ADC’s antibody has a higher affinity for HER2 than trastuzumab, which may increase HER2 selectivity and enhance its anti-tumor efficacy (Sheng et al., 2024). However, resistance to HER2-targeted therapies remains a significant hurdle, with both intrinsic and acquired resistances contributing to the failure of treatments in metastatic settings (Sarfaty and Rosenberg, 2020). Our findings suggest a correlation between LDH levels and HER2, indicating that HER2-positive tumors may rely more heavily on glycolysis. This dependency makes them viable targets for combined therapies that address both glycolytic pathways and HER2. Consequently, reassessing HER2 status and further clinical research is crucial to fully understand the benefits of combined HER2-targeted therapies in specific patient groups.

Furthermore, the variability in HER2 expression, alongside the possibility of intratumoral heterogeneity—indicated by differing HER2 scores within the same tumor—underscores the need for more precise biomarker assessment methods (Zhu et al., 2024). This could involve developing more sensitive and specific assays, as well as employing a combination of multiple biomarkers to enhance the accuracy of patient selection for targeted therapies. Additionally, the inconsistency between HER2 protein expression and gene amplification, as revealed by fluorescence *in situ* hybridization (FISH), underscores the complexity of evaluating HER2 in UC (Agersborg et al., 2018). This complexity suggests that a multimodal approach may be necessary to predict responses to HER2-targeted treatments accurately.

This study has several limitations that could affect the interpretation of its findings. The main limitation is the short follow-up period, which hinders our ability to thoroughly evaluate the impact of HER2 status on progression-free and overall survival. Understanding the long-term prognostic significance of HER2 positivity is essential for its clinical application, especially in developing personalized treatment plans. Future studies should aim to extend follow-up periods to better assess the effects of HER2 status on these critical survival metrics, thereby reinforcing its value as a prognostic marker and potentially guiding treatment decisions and patient management. Additionally, the small sample size of our study may not adequately represent demographic diversity, which might limit the generalizability of our results. The retrospective nature of the study also raises concerns about possible selection biases, and incomplete data collection could further complicate these issues.

## Conclusion

In conclusion, our study highlights the significant link between HER2 overexpression and both advanced tumor stages and increased LDH levels in UC. This underscores HER2’s crucial role in the disease’s progression and aggressiveness. We also identified a clear association between LDH levels and HER2 status, suggesting that HER2-positive tumors may rely more heavily on glycolysis. This metabolic reliance opens up avenues for combined therapeutic strategies that target both the glycolytic pathways and HER2 overexpression. Given HER2’s substantial impact on tumor dynamics, it is essential for future research to explore the underlying mechanisms of HER2-driven progression and to develop new combined therapeutic options.

## Data Availability

The original contributions presented in the study are included in the article/supplementary material, further inquiries can be directed to the corresponding author.
